# Foot pain and functional limitation in healthy adults with hallux valgus: a cross-sectional study

**DOI:** 10.1186/1471-2474-13-197

**Published:** 2012-10-16

**Authors:** Sheree E Nix, Bill T Vicenzino, Michelle D Smith

**Affiliations:** 1 Division of Physiotherapy, School of Health and Rehabilitation Sciences, The University of Queensland, St Lucia, 4072, QLD, Australia; 2 School of Clinical Sciences, Queensland University of Technology, Kelvin Grove, 4059, QLD, Australia

**Keywords:** Hallux valgus, Foot pain, Physical function, Footwear

## Abstract

**Background:**

Hallux valgus (HV) is a very common deformity of the first metatarsophalangeal joint that often requires surgical correction. However, the association between structural HV deformity and related foot pain and disability is unclear. Furthermore, no previous studies have investigated concerns about appearance and difficulty with footwear in a population with HV not seeking surgical correction. The aim of this cross-sectional study was to investigate foot pain, functional limitation, concern about appearance and difficulty with footwear in otherwise healthy adults with HV compared to controls.

**Methods:**

Thirty volunteers with HV (radiographic HV angle >15 degrees) and 30 matched controls were recruited for this study (50 women, 10 men; mean age 44.4 years, range 20 to 76 years). Differences between groups were examined for self-reported foot pain and disability, satisfaction with appearance, footwear difficulty, and pressure-pain threshold at the first metatarsophalangeal joint. Functional measures included balance tests, walking performance, and hallux muscle strength (abduction and plantarflexion). Mean differences (MD) and 95% confidence intervals (CI) were calculated.

**Results:**

All self-report measures showed that HV was associated with higher levels of foot pain and disability and significant concerns about appearance and footwear (p < 0.001). Lower pressure-pain threshold was measured at the medial first metatarsophalangeal joint in participants with HV (MD = −133.3 kPa, CI: -251.5 to −15.1). Participants with HV also showed reduced hallux plantarflexion strength (MD = −37.1 N, CI: -55.4 to −18.8) and abduction strength (MD = −9.8 N, CI: -15.6 to −4.0), and increased mediolateral sway when standing with both feet with eyes closed (MD = 0.34 cm, CI: 0.04 to 0.63).

**Conclusions:**

These findings show that HV negatively impacts on self-reported foot pain and function, and concerns about foot appearance and footwear in otherwise healthy adults. There was also evidence of impaired hallux muscle strength and increased postural sway in HV subjects compared to controls, although general physical functioning and participation in physical activity were not adversely affected.

## Background

Hallux valgus (HV) is a progressive foot deformity presenting with lateral deviation of the hallux and medial deviation of the first metatarsal head. Increasing HV severity is associated with subluxation of the first metatarsophalangeal joint (MTPJ) and presence of osteoarthritis (OA) [1]. HV is very common, affecting approximately 23% of adults [2], and surgical correction is frequently sought, making HV the most common indication for orthopaedic forefoot surgery [3].

While HV is basically regarded as a structural deformity, there is debate surrounding the association between abnormal foot structure and related pain and disability. Several studies in elderly populations have found no association between HV and self-reported foot pain and disability [4-9]; whereas, another study reported a significant association between HV and disabling foot pain in older adults [10]. Although fewer studies have investigated these associations in populations with a wide age range, recent studies have linked HV to greater self-reported foot pain and functional limitation in adults aged 40 to 69 years [11], and big toe pain in adults aged over 30 years [12]. Furthermore, there is evidence for impaired quality of life associated with HV and big toe pain in adults aged over 30 years [13], and poorer health-related quality of life and greater foot pain and disability with increasing HV severity in adults aged over 50 years [14].

In addition to self-reported pain and disability, the impact of HV on functional performance has been investigated with inconsistent findings. Impaired balance and gait patterns, toe muscle weakness, and an increased risk of falls in elderly individuals with moderate or severe HV have been reported by several studies [8,15-21]; however, other studies have shown no association between HV and postural sway [22], walking performance [4,6] or history of falls [5]. No previous studies have reported on physical measures of functional performance in adults of all ages with HV.

Two additional factors considered most important by patients and orthopaedic surgeons are concerns about cosmetic appearance and difficulty with footwear [23,24]. While a few population studies have investigated footwear factors such as past usage of high-heeled shoes [9] and shoe fit [5,25], no previous studies have investigated self-reported difficulty with footwear or concerns about foot appearance in individuals with HV not seeking surgical correction. Therefore, the primary aim of this cross-sectional study was to investigate differences in foot pain, functional limitation, concerns about appearance and difficulty with footwear in a group of adults with HV compared to matched controls. A secondary aim was to explore possible associations between these variables and severity of deformity in HV subjects.

## Methods

### Subjects

Thirty volunteers with HV (aged 20 years and older) and 30 controls matched for age (± 5 years), gender, and body mass index (BMI) (± 5 kg/m^2^) were recruited for participation in this study through community advertisements. Exclusion criteria were: history of foot or ankle surgery or fractures, hallux limitus (self-reported or first MTPJ passive dorsiflexion range of motion < 50 degrees) [26], inflammatory disease, neurological conditions, and a history of falls. Because radiographs were required to measure HV angle and determine inclusion into HV or control groups, pregnant or breastfeeding women were also excluded from this study. Weight-bearing dorsoplantar radiographs of both feet were obtained for all eligible participants by the same radiographer using a GE Definium 6000 Digital X-ray system. The HV angle (measured as the angle between the first metatarsal and proximal phalanx) [27] was determined from digital radiographs using computer software developed for telemedical applications [28], and HV cases were defined as an angle greater than 15 degrees. To be eligible for the control group, participants were required to have a radiographic HV angle less than 15 degrees on both feet. Radiographs were further examined by another examiner for signs of osteophytes and joint space narrowing with reference to a radiographic atlas developed by Menz et al. [29]. The case definition defined by these authors was used to classify cases of first MTPJ OA. Ethical approval was gained from the institutional Medical Research Ethics Committee, and all subjects gave written informed consent prior to participation.

### Measurement procedure

Height, weight and body mass index (BMI) were recorded in order to match HV participants with controls, and demographic data were obtained via questionnaire. All examinations and questionnaires were administered by a single examiner (SN). Intra-rater reliability for physical measures was determined from pilot work. Refer to Additional file 1: Table S1 for intraclass correlation coefficients (ICC_3,1_), standard error of measurement (SEM), and minimum detectable change at the 90% confidence limit. Reliability was considered good for ICCs greater than 0.75, and very good for ICCs greater than 0.9 [30].

### General health and physical activity

General health and well-being were assessed using the Short Form 36 Health Survey (SF-36v2®), which includes eight subscales [31]. Habitual physical activity levels were assessed using the Baecke Questionnaire [32] to calculate a work index, sport index, and leisure index.

### Self-reported foot pain and disability

To investigate functional disability related to foot pain, participants completed the Foot Health Status Questionnaire (FHSQ) [33] and Manchester Foot Pain and Disability Index (FPDI) [34]. The FHSQ contains four domains: foot pain (4 items), foot function (4 items), footwear (3 items), and general foot health (2 items). This questionnaire has been validated and shown to have good test-retest reliability [33]. FHSQ subscale scores are converted to a scale ranging from 0 to 100, with higher scores indicating better foot health. The original FPDI contained 19 items [34]; however, recent psychometric evaluation [35,36] has shown that 17 items cluster around three main constructs: functional limitation (10 items), pain intensity (5 items), and appearance (2 items). While different methods have been used for FPDI scoring [36], we used the approach described by Menz et al. [37]. Responses to questionnaire items were coded as follows: “none of the time” = 0, “some days” = 1, “on most/every day” = 2. A total score was calculated as the sum of item responses, resulting in an ordinal scale ranging from 0 to 34, and subscales were calculated for pain (score range 0 to 10) and function (score range 0 to 20).

Visual analogue scales (VAS) were used to investigate foot pain intensity and concerns about appearance. Worst and average foot pain intensity over the past four weeks were evaluated using a 100 mm VAS, with 0 mm described as “no pain” and 100 mm described as “worst pain ever,” and the pain location was identified on a foot diagram. The pain VAS has well-established reliability and validity in lower limb musculoskeletal research [38]. Participants were also asked to indicate how satisfied they were with the appearance of their feet on a 100 mm VAS, with 0 mm representing “completely satisfied with appearance” and 100 mm representing “completely dissatisfied with appearance.” This method was adapted from a technique described by Saro et al. [39].

### Footwear

Participants were asked to wear their regular footwear to the examination session. Shoes were assessed using a steel ruler to measure relative heel height (the difference between heel height and forefoot sole thickness) [25] and using digital callipers for relative ball width (the difference between forefoot width across the widest point of the MTPJs and the width of the shoe upper at the same point). A positive value for relative ball width indicated that the shoe upper was wider than the forefoot. Participants who wore sandals that were unable to be measured in this manner (n = 12) were excluded from this analysis. Finally, participants were asked whether they had ever regularly worn shoes with a heel height greater than two inches (yes/no), and how often they currently wore this style of shoe (never, seldom, sometimes, often, always) [25].

### Pressure-pain threshold

In order to obtain a quantifiable measurement of tenderness around the first MTPJ, as a surrogate for clinical palpation, pressure-pain threshold (PPT) was measured at the medial and plantar aspects of the joint. A digital pressure algometer (Somedic AB, Farsta, Sweden) was used to measure the pressure applied at a rate of 40kPa/s by a rubber-tipped probe (area 1cm^2^) [40]. An average of three measurements from each site was used for analysis.

### Functional performance and muscle strength

**Figure 1:**
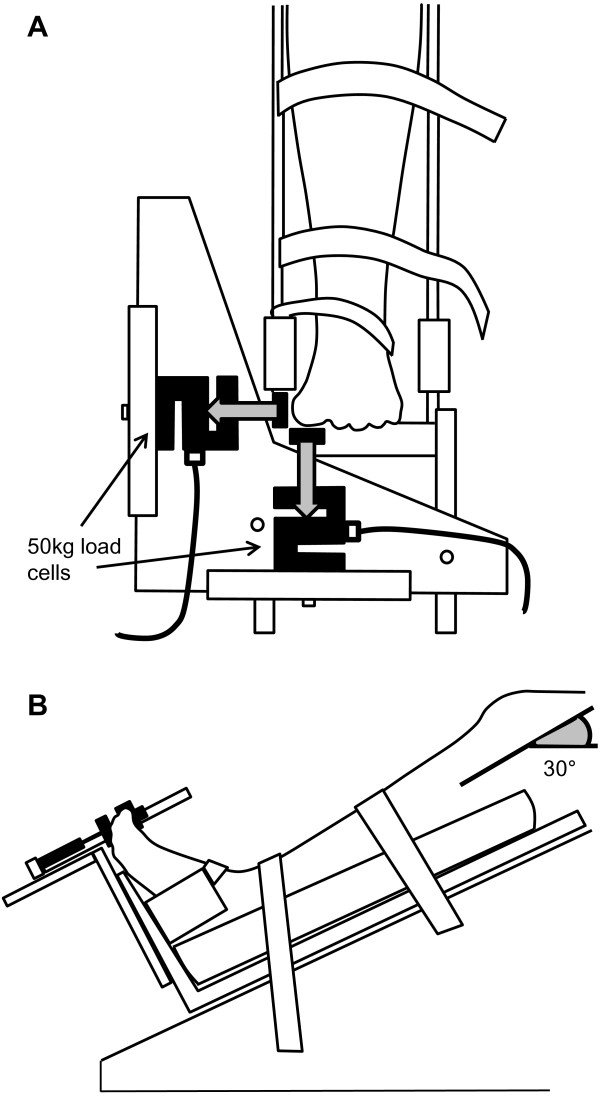
Design of custom load cell frame for testing hallux plantarflexion and abduction strength; A) dorsal/anterior view, B) lateral view.

Participants were asked to walk along a 10 metre walkway, and ascend and descend a set of 10 stairs (17.5cm high and 26cm deep) as quickly as possible. Each test was completed barefoot (without shoes or socks). The tests were recorded in seconds and the fastest of three trials was used for analysis. Similar functional performance tests have been used with good reliability in previous research [8]. Postural sway was examined using a force plate (Model 4060–07, Bertec, USA) and six different standing conditions: both feet on a firm surface with eyes open and closed, both feet on high-density foam (0.10 kg/cm^3^; 15 cm thickness) with eyes open and closed, and single leg stance on a firm surface with eyes open (left and right). A 70 second trial was completed for each condition [41]. If the subject was unable to complete the trial, a minimum of 30 seconds was required for the trial to be included in analysis. Data was analysed using Matlab (version 7.9; MathWorks, Natick, USA), and variables analysed were range of centre of pressure (COP) in both mediolateral and anteroposterior directions. Hallux plantarflexion and abduction strength were evaluated using 50 kg load cells (GK 2126–50, Gedge Systems, Melbourne, Australia) mounted in a custom-built frame (Figure 1). The load cells were calibrated prior to each measurement session, and the signal converted to force (N). Participants were seated with the knee in 30 degrees of flexion and the lower leg and foot stabilised using Velcro straps. After familiarisation with the required movements, participants were asked to perform three maximum isometric voluntary contractions in hallux plantarflexion and abduction. The examiner ensured that the subject’s heel remained in contact with the base of the frame and that there was minimal activity of lower leg muscles. The maximum force achieved over three trials was used for analysis.

### Sample size determination

Sample size was based on a priori power calculations. Using standard deviations obtained from preliminary data analysis (n = 26), we determined that 29 subjects in each group would provide 80% power to detect a difference of 11 mm between groups on the 100 mm pain VAS (alpha 0.05) [42,43].

### Statistical analysis

Age and BMI were first compared using independent t-tests to ensure that there were no significant differences between HV and control groups. For variables measured bilaterally (appearance VAS, hallux strength, PPT, and postural sway in single leg stance), data from only one limb was analysed [44]. For HV subjects the foot with a greater HV angle was chosen (16 right feet and 14 left feet used for analysis), while for control subjects the right or left foot was chosen at random using a random number generator (15 right feet and 15 left feet used for analysis). All variables were examined for normality of distribution using boxplots, histograms, and quantile-normal plots. Continuous variables showing a skewed distribution were transformed wherever possible using log, square root, or inverse square root transformations, as appropriate. Differences between groups were then examined using independent t-tests for continuous variables, Wilcoxon rank-sum tests for ordinal variables and continuous variables for which no adequate transformation was available, and Chi-squared statistics for categorical data. In the HV group (n = 30), the relationship between HV angle and other variables was investigated using Spearman’s correlation coefficient. Correlations are reported for variables showing statistically significant correlations with HV angle. Spearman’s rho was interpreted as follows: low correlation (rho = 0.26 to 0.49), moderate correlation (rho = 0.5 to 0.69) or high correlation (rho > 0.7) [45]. Results for continuous variables are presented as means, standard deviations (SD), mean differences (MD), and 95% confidence intervals (CI), while ordinal data are presented as medians (min – max). All analyses were conducted using STATA version 10 [46], and the level of significance was set at p < 0.05.

## Results

### Subjects

Mean age of the total sample was 44.4 years (SD 15.1, range 20 to 76 years) and mean BMI was 24.5 kg/m^2^ (SD 3.8, range 18.0 to 35.4 kg/m^2^), with HV and control groups matched on these characteristics. The mean absolute difference between matched subjects for age was 1.6 years and for BMI was 1.7kg/m^2^. There were 25 women and five men in each group. Subject characteristics, as well as radiographic HV angle and presence of first MTPJ OA in each group are presented in Table 1.

**Table 1:** Characteristics of HV and control groups with comparison of SF-36v2® subscales and physical activity levels (mean ± SD, unless otherwise stated)

	**HV group (n = 30)**	**Control group (n = 30)**	**Mean difference (95% CI)**
Subject characteristics			
Men/Women (n)	5/25	5/25	NA
Age (years)	44.5±15.2	44.2±15.3	0.3 (−7.6 to 8.2)
BMI (kg/m^2^)	24.2±3.4	24.7±4.2	−0.5 (−2.5 to 1.5)
HV angle (°)†	29.1±7.8	9.8±3.5	19.3 (16.2 to 22.4)**
First MTPJ OA (n (right/left))	1 (1/1)	2 (2/1)	NA
SF-36v2® (score range 0 – 100)			
Physical functioning	86.6±19.3	93.2±9.7	−6.6 (−14.5 to 1.3)
Role-physical	87.5±17.9	93.3±13.7	−5.8 (−14.1 to 2.4)
Role-emotional	93.1±11.4	93.9±11.1	−0.83 (−6.7 to 5.0)
Bodily pain	75.0±18.8	81.8±14.0	−6.8 (−15.4 to 1.8)
Vitality	59.4±17.3	67.1±12.2	−7.7 (−15.6 to 0.11)
Mental health	79.7±10.8	83.5±11.0	−3.9 (−9.5 to 1.8)
Social functioning	91.3±12.8	89.2±15.3	2.1 (−5.2 to 9.4)
General health	78.5±17.2	78.8±13.1	−0.3 (−8.2 to 7.6)
Physical activity (score range 1 – 5)			
Work	2.62±0.33	2.45±0.32	0.17 (0.0 to 0.34)*
Sport	2.92±0.94	2.23±0.65	0.69 (0.28 to 1.1)*
Leisure	2.87±0.63	2.88±0.55	−0.01 (−0.32 to 0.30)

Abbreviations: HV = hallux valgus; CI = confidence interval; BMI = body mass index; MTPJ = metatarsophalangeal joint; OA = osteoarthritis; SF-36v2® = Short Form 36 Health Survey, version 2.

* *P* < 0.05.

** *P* < 0.001.

† Only one foot included in analysis (HV group: worst foot; control group: randomly chosen).

### General health and physical activity

There were no significant differences between HV and control groups for SF-36v2® subscales, although the difference between groups on the vitality subscale (MD = −7.7, CI: -15.6 to 0.11) approached statistical significance (p = 0.05). HV participants reported significantly higher sporting activity (MD = 0.69, CI: 0.28 to 1.1) and physical activity levels at work (MD = 0.17, CI: 0.0 to 0.33), while the leisure index was not significantly different between groups (Table 1).

### Foot pain and disability

Significant between-group differences were found for all measures of self-reported foot pain and disability, including FHSQ and FPDI subscales (Table 2). Participants with HV reported more foot pain over the past four weeks on a 100mm VAS for worst pain (MD = 25.5 mm, CI: 14.3 to 36.6) and average pain (MD = 12.3 mm, CI: 6.2 to 18.3). PPT was lower at the medial first MTPJ in participants with HV (MD = −133.3 kPa, CI: -251.5 to −15.1), although there was no significant difference in PPT at the plantar first MTPJ.

**Table 2:** Comparison between HV and control groups for self-reported foot pain, disability and concern about appearance (mean ± SD for all tests except FPDI presented as median (min – max))

	**HV group (n = 30)**	**Control group (n = 30)**	**Mean difference (95% CI)**
FHSQ (score range 0 – 100)			
Foot pain scale	76.8±18.5	94.9±6.2	−18.1 (−25.3 to −11.0)**
Foot function scale	86.9±17.7	99.4±2.5	−12.5 (−19.0 to −5.9)**
Footwear scale	28.1±23.1	75.5±25.4	−47.5 (−60.0 to −34.9)**
General foot health	49.1±29.0	86.6±13.9	−37.5 (−49.3 to −25.7)**
VAS (0 – 100 mm)			
Worst pain VAS	37.6±25.5	12.2±16.9	25.5 (14.3 to 36.6)**
Average pain VAS	15.4±15.1	3.2±6.9	12.3 (6.2 to 18.3)**
Appearance VAS	58.4±31.4	20.4±23.2	38.1 (23.8 to 52.3)**
Pressure-pain threshold (kPa)			
Medial first MTPJ	561.6±224.1	694.9±233.3	−133.3 (−251.5 to −15.1)*
Plantar first MTPJ	448.7±196.8	539.5±231.5	−90.8 (−201.8 to 20.3)
Footwear examination (mm)			
Relative heel height	15.2±11.8	16.2±12.3	−0.96 (−7.3 to 5.4)
Relative ball width†	4.6±4.6	4.8±5.2	−0.22 (−3.1 to 2.6)
FPDI			Wilcoxon rank-sum Z
Function (score range 0 – 20)	2 (0 – 14)	0 (0 – 5)	−4.88**
Pain (score range 0 – 10)	3 (0 – 8)	0 (0 – 2)	−5.81**
Total (score range 0 – 34)	5.5 (0 – 24)	0 (0 – 9)	−6.18**

Abbreviations: HV = hallux valgus; CI = confidence interval; FHSQ = Foot Health Status Questionnaire; VAS = Visual analogue scale; MTPJ = metatarsophalangeal joint; FPDI = Foot Pain and Disability Index.

* *P* < 0.05.

** *P* < 0.001.

† Twelve subjects were excluded from this analysis; therefore analysis was based on n = 48 (23 HV, 25 control).

Analysis of foot pain locations showed that 25 HV subjects (83%) reported pain in the first MTPJ or hallux, eight subjects (27%) reported pain in the lesser toes or MTPJs, and six subjects (20%) reported pain in the heel or midfoot area. There were 12 HV subjects who reported foot pain in more than one location, and only two who reported no foot pain. Control subjects reported the following distribution of foot pain: pain under the first MTPJ (one subject, 3%), pain in the lesser toes or MTPJs (nine subjects, 30%), and pain in the heel or midfoot (13 subjects, 43%). Three control subjects reported pain in more than one location, and nine reported no foot pain.

### Concerns about appearance and footwear

Participants with HV had significant concerns about foot appearance (VAS: MD = 38.1 mm, CI: 23.8 to 52.3) and more difficulty fitting footwear (FHSQ footwear score: MD = −47.5, CI: -60.0 to −34.9) than control subjects. On the FPDI item which states “I feel self-conscious about my feet”, 19 participants with HV (63%) responded “on some days” or “on most/every day”, compared to five participants (17%) in the control group (Chi-squared p = 0.001). Similarly, 13 HV participants (43%) responded positively to the statement “I get self conscious about the shoes I have to wear”, compared to one participant (3%) in the control group (Chi-squared p = 0.001). Fifteen participants in the HV group and 16 control participants reported a history of regularly wearing high heeled shoes (> 2 inches). Eight participants in each group reported wearing high heels “sometimes,” while high heels were worn “often” by four control participants and two HV participants, and “always” by one person with HV. Examination of footwear worn to the examination session showed no significant differences between groups in relative heel height or relative ball width measures (p > 0.05) (Table 2).

### Functional performance and muscle strength

**Figure 2:**
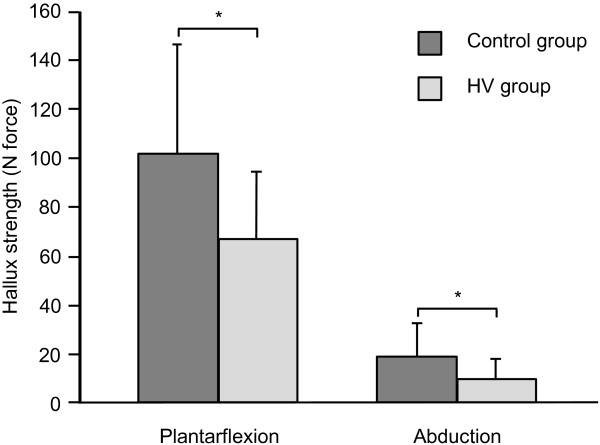
Hallux plantarflexion and abduction strength for HV and control groups; * indicates a significant difference between groups (p < 0.001).

Functional performance measures are presented in Table 3. There were no significant differences in walking performance between groups (p > 0.05). Mediolateral sway while in double-leg stance on a firm surface with eyes closed was the only postural sway parameter that was different between groups, with a significant increase in mediolateral COP range in the HV group compared to controls (p = 0.03). In single leg stance, seven subjects (4 HV, 3 controls) were unable to complete the entire 70-second trial, and two of these trials (< 30 sec) were excluded from analysis. As shown in Figure 2, the HV group had significantly weaker hallux plantarflexion strength (MD = −37.1 N, CI: -55.4 to −18.8) and hallux abduction strength (MD = −9.8 N, CI: -15.6 to −4.0) compared to controls.

**Table 3:** Comparison between HV and control groups for functional performance and muscle strength (mean ± SD)

	**HV group (n = 30)**	**Control group (n = 30)**	**Mean difference (95% CI)**
Walking tests (sec)			
Timed 10m walk	4.95±0.69	4.99±0.59	−0.04 (−0.37 to 0.29)
Stair ascent	3.88±0.41	3.77±0.41	0.11 (−0.11 to 0.32)
Stair descent	3.58±0.45	3.46±0.44	0.12 (−0.11 to 0.35)
Postural sway (COP range, cm)			
AP sway, both feet eyes open	1.91±0.82	1.90±0.62	0.0 (−0.37 to 0.38)
ML sway, both feet eyes open	1.50±0.65	1.35±0.40	0.15 (−0.13 to 0.43)
AP sway, both feet eyes closed	2.13±1.4	2.08±0.97	0.05 (−0.58 to 0.68)
ML sway, both feet eyes closed	1.78±0.68	1.44±0.46	0.34 (0.04 to 0.63)*
AP sway, foam eyes open	3.06±1.2	2.78±0.82	0.28 (−0.26 to 0.82)
ML sway, foam eyes open	3.63±1.1	3.43±0.91	0.20 (−0.33 to 0.73)
AP sway, foam eyes closed	5.94±3.3	4.87±1.4	1.1 (−0.24 to 2.4)
ML sway, foam eyes closed	7.80±2.9	6.61±1.8	1.2 (−0.06 to 2.4)
AP sway, single leg stance†	3.44±1.2	2.97±0.85	0.47 (−0.07 to 1.0)
ML sway, single leg stance†	5.54±2.8	4.56±1.4	0.98 (−0.16 to 2.1)
Muscle strength (N)			
Plantarflexion strength	66.9±29.0	104.0±40.7	−37.1 (−55.4 to −18.8)**
Abduction strength	9.9±7.7	19.7±13.9	−9.8 (−15.6 to −4.0)**

Abbreviations: HV = hallux valgus; CI = confidence interval; COP = centre of pressure; AP = anteroposterior; ML = mediolateral.

* *P* < 0.05.

** *P* < 0.001.

† Two excluded trials (< 30 sec) in HV group; therefore analysis was based on n = 58.

### Correlations with HV angle

A significant inverse correlation was found between greater HV angles and lower FHSQ scores indicating poorer general foot health (rho = −0.41, p = 0.03). Greater HV angles were correlated with higher appearance VAS scores indicating less satisfaction with appearance (rho = 0.43, p = 0.02). There was also a significant inverse correlation between greater HV severity and poorer hallux abduction strength (rho = −0.41, p = 0.03). Across the SF-36v2® subscales, low correlations were found between HV angle and bodily pain (rho = 0.39, p = 0.04) and social functioning (rho = 0.49, p = 0.01), indicating that in our sample, HV subjects with more severe deformity reported less bodily pain and better overall social functioning. No significant correlations were found for other SF-36v2® subscales, and HV angle was not correlated with footwear difficulty or any other measure of foot pain and function (i.e. FHSQ, FPDI, VAS, PPT).

## Discussion

This study investigated self-reported foot pain and disability, functional performance, concerns about appearance and difficulty with footwear experienced by healthy adults with HV. We secondarily explored possible associations between severity of HV angle and other variables in participants with HV.

Our results show that the presence of HV deformity is associated with self-reported foot pain and disability. Significant differences were found between HV and control groups for FHSQ and FPDI subscales, as well as worst and average pain VAS (Table 2). With reference to the minimal important difference for the FHSQ and VAS previously reported by Landorf et al. [43], these differences between groups can be considered clinically meaningful. Our findings are similar to those reported by Cho et al. [11] who showed that HV was associated with more self-reported foot pain and poorer self-reported physical functioning in adults aged 40 years and older (n = 563). Abhishek et al. [13] further highlighted the importance of big toe pain accompanying HV, reporting that health-related quality of life was progressively impaired in adults aged 30 years and older (n = 3082) with HV alone, big toe pain alone, and HV with big toe pain. In our study there was also some evidence of mechanical hyperalgesia around the medial aspect of the first MTPJ, as indicated by a lower PPT in HV subjects (p < 0.05). However, this result should be interpreted with caution as the difference between groups (MD = −133.3, CI: -251.5 to −15.1) did not reach the calculated MDC_90_ (255.2) (Additional file 1: Table S1) and therefore may not represent a meaningful difference.

In addition to self-reported foot pain and functional limitation, participants with HV in our study presented with hallux plantarflexion and abduction weakness and increased mediolateral postural sway. Our finding of decreased hallux plantarflexion strength in people with HV is consistent with that of Mickle et al. [21]. However, our study also showed a significant inverse correlation between HV angle and an individual’s ability to abduct the hallux (rho = −0.41), a finding which has been suggested by electromyographic investigations [47,48] but previously has not been investigated clinically. With regard to standing balance, results reported by Mickle et al. [22] are in contrast to our findings, as these authors reported no difference in postural sway between older adults with HV and controls. However, other studies in elderly populations have found poorer lateral stability, poorer coordinated stability, and increased postural sway to be associated with HV [17,19]. Finally, no significant between-group differences were found in walking performance in our study, which is consistent with previous findings in elderly populations [4,5,8,19].

Despite no differences between groups in relative heel height or relative ball width of footwear worn to the examination, participants with HV reported significantly more difficulty with footwear and concerns about foot appearance than controls. Concerns about appearance and general foot health appeared to increase with severity of deformity in the HV group (appearance VAS: rho = 0.43, FHSQ general foot health: rho = −0.41). To our knowledge, this is the first study to investigate self-reported difficulty with footwear and concerns about appearance in a HV population not seeking surgical correction compared to age and gender-matched controls. Our data suggest that clinicians managing HV should place particular priority on footwear concerns. This is supported by Saro et al. [24], who showed that free choice of footwear was significantly associated with health-related quality of life outcomes after HV surgery.

While participants with HV in our study reported significantly more foot-specific functional disability, participation in physical activities, general health and physical functioning were not adversely affected (Table 1). To the contrary HV subjects reported higher levels of habitual physical activity at work and in sports, which may have led to improved performance on other physical parameters such as walking and balance tests. Furthermore, correlations between HV angle and SF-36v2® subscales suggest that in our sample HV participants with more severe deformity experienced less bodily pain and better social functioning. This finding is in contrast to results of a recent study by Menz et al. [14] who found a trend towards poorer scores on all SF-36v2® subscales with increasing HV severity in adults aged 50 years and older (n = 2681). It is possible that general health and functioning may be more impacted by increasing HV severity in populations of older adults; alternatively, it may be that our findings were a consequence of a volunteer sample of HV participants, who were active individuals with a high level of physical functioning. Furthermore, perhaps those with more severe HV in our sample had adapted their lifestyle or footwear choices to accommodate for a severe foot deformity.

Caution must be applied when comparing reports from different studies as varying case definitions (present/absent or mild/moderate/severe) and means of diagnosing HV (i.e. self-reported or diagnosed by an examiner) are used [2]. Studies have used a range of methods to evaluate the presence and severity of HV, including weight-bearing radiographs [11,49], and the Manchester Scale [10,17,19,22,50], which includes a series of four standardised photographs used by an examiner to classify HV as “none,” “mild,” “moderate,” or “severe.” Other larger studies have used a validated self-report instrument based on five line drawings representing increasing HV severity [12-14,51]. Our study used standardised weight-bearing radiographs and a widely accepted angular definition to classify HV as being present (HV angle > 15°) [52]. This definition meant that several mild and asymptomatic HV cases were included, which was considered appropriate to address the primary research question of whether the presence of HV was associated with foot pain and disability.

Our study findings should be interpreted with consideration of our recruitment methods and sample, which may affect the generalisability of results. First, volunteers with HV responding to advertisements were likely aware that they had a foot problem. This may have introduced an element of bias to their self-reported foot health measures compared to controls. Second, only ten males participated in this study, and the age range of study participants was relatively wide. Nevertheless, these sample characteristics were considered representative of a clinical population. Third, whilst our sample included participants with mild, moderate and severe HV, the sample size in the current study was not sufficient to examine subgroups according to HV severity. While there is some evidence that increasing HV severity has a greater impact on foot pain and disability [14], further research using large population-based samples is warranted to determine whether HV severity is associated with increased foot pain or poorer functional performance.

Reliability of measurement methods must be considered as a potential limitation in any clinical research. All measurements in our study were performed by the same examiner, and intra-rater reliability was very good for most physical measurements (Additional file 1: Table S1). Inter-rater reliability was not addressed by this investigation. Reported intra-rater reliability for hallux plantarflexion and abduction strength was lower than desirable (ICC_3,1_ = 0.73 to 0.75), and as a result the calculated values for MDC_90_ (plantarflexion: 47.8 N; abduction: 14.5 N) were quite large for these measures. While the differences between HV and control groups were statistically significant (plantarflexion: MD = −37.1 N; abduction: -9.8 N), the clinical significance of these results should be interpreted with caution. Methods previously reported to measure hallux plantarflexion strength include the clinical paper grip test [53], strain gauge scales [54], force plate [55] or pressure platform systems [21]. We developed a novel method that would not only give a continuous-scaled quantitative measurement, but would also allow us to examine participants’ ability to abduct the hallux.

Finally, some discussion is warranted regarding self-report data obtained in our study. Both foot-specific questionnaires (FHSQ, FPDI) produced significantly skewed data, and consequently non-parametric statistical tests were used. In particular the summed FPDI scores cannot be interpreted as a true interval scale unless a Rasch analysis is performed [36], which was not undertaken for the current study. Potential for recall bias and variation between individuals’ interpretation of pain rating scales should also be considered when interpreting self-report data [56,57]. It is interesting to note that self-report measures of foot-specific pain and disability showed large differences between groups, while self-reported general functioning and physical performance tests were less indicative of limitations in participants with HV. Study participants reported more pain and disability than was evident on physical performance tests, thus it may be that foot-specific questionnaires (FHSQ, FPDI) capture more than typical measures used for physical function. For example, while general physical functioning (SF-36v2®), activity participation and walking performance were not impaired in this sample, HV subjects reported significant functional disability on the FPDI, which considers the influence of foot pain and other aspects such as walking distance and rough or hard surfaces. Future studies could utilise more challenging physical performance tasks to explore functional difficulty experienced by healthy adults with HV.

## Conclusion

HV deformity is accompanied by significant foot-specific pain and disability, muscle weakness around the first MTPJ and increased mediolateral postural sway. Concerns regarding appearance and footwear are also important factors to consider for clinicians managing this common deformity. Global physical functioning and participation in activities were not adversely affected in our sample, indicating that HV may not prevent participation in an active lifestyle in otherwise healthy adults.

## Abbreviations

BMI: Body mass index; CI: 95% confidence interval; COP: Centre of pressure; FHSQ: Foot Health Status Questionnaire; FPDI: Manchester Foot Pain and Disability Index; HV: Hallux valgus; ICC_3,1_: Intraclass correlation coefficient (3,1); MD: Mean difference; MDC_90_: Minimal detectable change (90% confidence limit); MTPJ: Metatarsophalangeal joint; OA: Osteoarthritis; PPT: Pressure-pain threshold; SD: Standard deviation; SEM: Standard error of measurement; SF-36v2®: Short Form 36 Health Survey version 2; VAS: Visual analogue scale.

## Competing interests

The authors declare that they have no competing interests.

## Authors’ contributions

All authors (SN, BV, MS) made substantial contributions to the conception and design of the study, interpretation of data, and critical revision of the manuscript. SN performed data acquisition and analysis and prepared the manuscript. All authors read and approved the final manuscript.

## Pre-publication history

The pre-publication history for this paper can be accessed here:

http://www.biomedcentral.com/1471-2474/13/197/prepub

## Supplementary Material

Additional file 1 **Table S1.** Test-retest reliability for physical measures. Click here for file
